# Gray and Green Revisited: A Multidisciplinary Perspective of Gardens, Gardening, and the Aging Process

**DOI:** 10.1155/2014/283682

**Published:** 2014-03-06

**Authors:** Scott D. Wright, Amy Maida Wadsworth

**Affiliations:** ^1^Gerontology Interdisciplinary Program, University of Utah, Salt Lake City, UT 84112-5880, USA; ^2^Family and Consumer Studies Department, University of Utah, Salt Lake City, UT 84112, USA

## Abstract

Over fourteen years ago, the concept of “gray and green” was first introduced by Wright and Lund (2000) to represent a new awareness and a call for increased scholarship at the intersection of environmental issues and the aging process. This review paper revisits that concept with a fresh perspective on the specific role of gardens and gardening in the aging experience. As example, gardening is one of the most popular home-based leisure activities in the US and represents an important activity in the lives of older adults in a variety of residential settings. Yet, there has been a lack of any comprehensive and multidisciplinary (science and humanities) examination of the nexus between gardening and the aging experience, and in particular with research connections to stewardship and caring. In this paper, we review contemporary articles demonstrating the multidisciplinarity of gardening and the aging process. First, we will focus on the beneficial psychological effects resulting from the cultivation of caring, including personal contentment and artistic expression. Second, we will focus on stewardship and how gardening increases health, community awareness, and a connection to future generations. On the surface, this may demonstrate a separation between the humanities and science, but we will clarify a symbiotic relationship between the two disciplines in our conclusion.

## 1. Introduction

Over fourteen years ago, the concept of “gray and green” was first introduced by Wright and Lund [1] as representing new awareness and call for increased scholarships at the intersection of environmental issues and the aging process [2–4]. An important dimension of that original article [1] was to bring more attention to the role of the natural environment in contrast to the “built environment” in relation to successful aging [5]. This review paper revisits the concept of *gray and green* with a fresh perspective using selected examples from the literature on the specific role of gardens and gardening as it enhances the aging experience. Although the examination of the horticultural and human experience has been previously examined, especially in the context of well-being and social development [6, 7] and in the lives of the elderly [8], this review paper offers a more contemporary perspective on the increasing number of publications since the early 1990s that have addressed gardening, gardens, and aging, particularly since the emergence of the “gray and green” publication in the year 2000 [1].

Gardening is one of the most popular home-based leisure activities in the US and represents a significant and salient activity in the lives of older adults [9–15]. The National Gardening Association claimed that 80% of US households tended to plants, which represented an increase of 65% from the year 1996 [16]. The 55- to 64-year-old age group was the cohort that spent the most on horticultural products and services and this trend will most likely continue with aging baby-boomers [16, 17]. Some have proposed that there is an increased return to the small-scale farming approach as a new calling in life for many aging individuals. This trend is supported by the growing interest in the organic food movement for both environmental and health-conscious ideals [18].

However, it is proposed in this paper that gardening may represent a facet of a much deeper gerontological connection between the natural environment, well-being, legacy, sense of meaning, and interpersonal connections to community and larger social structures [12]. The functional allure of gardens and gardening in the later years is the result of multiple factors: historical, aesthetic, cultural, generational, psychological, and physiological factors. It is the premise of this paper that there is much more to the nexus of *gardening and aging* than a favorite pastime, hobby, or a task aligned with passive drudgery. For example, in the book, *Gardening for a Lifetime: How to Garden Wiser as You Grow Older*, the author Eddison [19] believes gardening can become a joy-filled activity in later life even with the onset of comorbidities and chronic conditions associated with advancing age, if the gardener considers adopting wise alternatives to high maintenance practices in the garden. Gardening and gardens represent an important facet of the *gray and green* concept where the interaction with horticulture and landscaping is a holistic experience for a “mind-body-spirit” connection [15]. This interactive connection is further highlighted by representative books such as Patricia Cassidy's [20] *The Age Proof Garden* and *The Illustrated Practical Guide to Gardening for Seniors: How to maintain your outside space with ease into retirement and beyond, *where the focus is on gardening and the aging experience, but as an adaptive process that understands and appreciates the benefits of gardening, but with adjustments in maintenance and goals in the older adult's garden.

In this paper, we review contemporary articles demonstrating the multidisciplinarity of gardening and the aging process. First, we will focus on the beneficial psychological effects resulting from the cultivation of caring, including personal contentment and artistic expression. To do this, we will reference contemporary editions of classic texts to explore the still-relevant implications of historical gardening themes and allegories. We will also discuss aging painters, writers, and filmmakers to demonstrate how the theme of gardening permeates the aging experience in various expressions of human adaptation, creativity, and existential significance even in the face of challenge and loss.

Second, we will focus on stewardship and how gardening increases health, community awareness, and represents a connection with future generations. Most importantly, we advocate the premise that gardens and gardening represent an important bridge to connect the aging experience to the natural world where stewardship, caring, and well-being are realized.

On the surface, this multidisciplinary focus may demonstrate affinity for C.P. Snow's *Two Cultures* and a separation between the humanities and science, but we will clarify a symbiotic relationship between the two disciplines in our conclusion.

## 2. Process and Procedure for the Selected Literature Review

The literature was systematically reviewed for relevant and peer-reviewed journals, books, and book chapters as well as contemporary annotated releases of classic and modern literature such as Thoreau's *Walden* and Virgil's *Georgics*. Relevant books were identified through published scholarly journal book reviews, published journal article references, and Google Scholar and through the Amazon.com search tool. To generate a list of selected articles (as of January 12, 2014) for the paper, searches were done in four electronic databases: PsychINFO, PubMed, ProQuest, and Scopus. The search strategy was complemented by inspecting the included articles and their reference lists. The tactical goal was to use PsychINFO, PubMed, ProQuest, and Scopus as the databases to conduct searches in the literature and establish a convergence of relevant publications from multiple database search tools. The review was primarily grounded in a Scopus query algorithm to establish corroboration of relevant publications and to generate analytics for the primary search terms (*gardening and aging*).Scopus is a large abstract and citation database of research literature and web sources representing 50 million records, 21,000 titles, and 5,000 publishers. The search using Scopus was conducted through a university system (retrieved January 12, 2014) and it is considered to have a robust index and coverage of publications. The Scopus search for (gardening AND aging) in ALL fields in journals articles published or in press and limited to a Date Range from 1990 to Present resulted in 492 document results and the analytics revealed a modest increase in the total number of documents for this query by year since 1990 to the search in January 12, 2014. When using the limiter “Article title, Abstract, Keywords” (i.e., TITLE-ABS-KEY) default in the query, the search resulted in 66 document results. A thorough examination was conducted of the 66 documents retrieved from Scopus and then articles were selected for relevancy and content affinity to the main themes of this paper.

## 3. How Does Your Garden Grow? Definitions, History, and Purposes

For many people, the word “garden” can evoke varied emotions and leaps of cognitive associations. According to the Oxford English Dictionary (OED), “garden” can be approached and treated as a noun and defined as, “a piece of ground adjoining a house, used for growing flowers, fruits, or vegetables,” and as a modifier such that, gardens can represent “ornamental grounds laid out for public enjoyment and recreation.” [21]. The OED also recognizes that one can become an active participant by working in a garden as a “gardener” (noun), by having “gardened” (verb). These distinctions serve as a basis for the reader to consider the intersection of the aging process with gardens and gardening, which can be active and passive, functional and aesthetic, and subjective and objective. For example, the term, “garden” may signify many things that have little to do with cultivating vegetables and flowers and instead, as example, focus on landscaping as gardening *with nature* [22]. Or some may immediately think of an entertainment/sports venue (e.g., Madison Square Garden); a biblical setting (e.g., The Garden of Eden) or some historical wonder of the world (e.g., The Hanging Gardens of Babylon); or pieces of literature such as *The Garden of Forking Paths* by Jorge Luis Borges or the film *The Constant Gardener *directed by Fernando Meirelles (based on the novel by John le Carre); or exotic paintings like *The Garden of Earthly Delights* by Hieronymus Bosch. For others, “garden” may represent the Butchart Gardens in Greater Victoria on Vancouver Island, Canada, or Zen rock garden of the Ryoanji Temple in Kyoto, Japan [23–25]. In the sociological domain, gardens have served as a focal point for assessing collectivist and bureaucratic cultures in context of the “urban gardening movement” [26], and more recently as a practice to engage in the illicit cultivation of someone else's land known as “guerrilla gardening” [27]. In the historical domain, there were the “Victory Gardens” in the 1940s and with contemporary television, perhaps the PBS series, “The Victory Garden.” But for many people, a garden can simply be a plot of soil as close as your backyard and as modest as raised box with a few marigolds and tomato plants. Thus, as Ross [28–39] has noted, trying to pinpoint an exact definition of gardens is daunting given the kaleidoscopic variations of gardens across many cultures and geographies. For example, Pizzoni [30] has advocated that “*gardens can be thought of as a place set aside for multiple uses such associated with horticulture and the cultivation of plants for food and medicinal herbs but it can also be seen as an expression of ornamental, religious and even political purposes*”(see also [12, 31–40] for further discussion). In this paper, we will advance a similar understanding of gardens and gardening and the aging experience but broaden the horizon such that health and well-being can be derived as a benefit, and this includes the psychological expression in our main premise: *to be generative, to cultivate, to care is both utilitarian and sacred and finds its joyful expression in the reverential duty in the garden.*

### 3.1. Humanities Discipline: How Gardens and Gardening Cultivate Caring and a Personal Connection with Life and Nature

The word “garden” may conjure memories of warm, fertile dirt in your hands, the smell of blossoms, the sound of bees collecting pollen, or the beautiful, flourishing sight of what humans and nature can accomplish together. Gardens are experienced through the senses, and therefore, the psychological assimilation of a garden and all it may represent is highly personal. It is no wonder, then, that a deep personal understanding of life results from such communion with nature and may translate into the expression of self through art.

For example, the interrelationship between painting and the use of garden as motif or actual object for inspiration represents an important, intense, and intimate thread in the history of art and reinforces the sensory connection between human and garden. There is perhaps the most spectacular and enigmatic work of Hieronymus Bosch and his haunting imagery associated with the triptych, *Garden of Earthly Delights*. *The Garden of Earthly Delights* was painted around the years 1503 and 1504 when Bosch was about 50 years of age [41], although he lived well into the mid-sixties of life. *Garden of Earthly Delights* is the middle panel of the triptych and captures sinful pleasures in a garden-like setting where naked figures parade about and into pools of water which can bring to mind primeval fountains of youth. Compared to the panel “Paradise” which depicts a more balanced ecosystem of sorts, the middle panel is awash in people, and the garden appears to be magically self-sustaining with little human activity involved in maintenance or cultivating its rich resources for all. And indeed, there are no children to be found in this Arcadian landscape and everyone appears to be *ageless*. This is a presentation of a carefree world where the garden serves as a symbolic Utopia and humankind resides in paradise untouched by the “Fall” [41]. Of course, it is the other panel, *Hell*, which exacts the most hallucinatory experience and presentation of nightmarish figures. Perhaps, we can think of that desperate landscape, like T.S. Eliot's waste land, as a reminder of where there are repercussions for engaging *careless* activities that did not take into account the stewardship clause when residing in the Garden of Eden. We reap what we sow, but in this case the “Fall” may represent itself when all was spent in the present and little effort was taken to cultivate for the next season, or the next generation, thus leading to the hellish demise of humanity.

In direct contrast is the work of Claude Monet, who is considered as one of the founders of style of painting known as French Impressionism, lived a remarkable eighty-six years of life that spanned across two centuries (from 1840 to 1926). Monet proved to be extremely productive with creating some of his most famous paintings into his later years and along with other many other painters (e.g., Henri Matisse, Rembrandt, and Georgia O'Keefe) he has been the subject of research examining creative and artistic style and productivity into the second half of life [42–46].

Many people are certainly aware of the vast array of Monet's work and many can also readily identify the unique style and composition of his paintings, such that, for example, when examining *water lilies* with rich textures or admiring *irises* with saturated colors, we come to think that those flowering plants are virtually synonymous with *his *portfolio of art. But then again many people may *not *know that those paintings were created based on the living landscape of his own home and garden located in Giverny, France. Monet was both painter *and* gardener and he spent the last 43 years of his long life with his close-knit family by cultivating his passion for creating color with flowering plants on a one-hectare (2.5 acre) garden site and then capturing the dynamic and fluid presence of those plants onto his canvas with brushwork and oils [45]. But his garden would also be a healing and therapeutic oasis that helped him cope and adjust to the loss of his second wife and his son Jean. Monet was reported as saying, “*My most important work of art is my garden*” [47]. From 1908 and at the age of 68, Monet focused his artistic work almost entirely on depicting his garden and was then commissioned by the state to create a remarkable large format series of paintings of water-lilies (*Grandes Decorations*), his tour de force, that formed an enveloping circle that was to reside in a specially constructed pavilion in the Musée de l Orangerie, which was an extension to the Louvre [47, 48]. Around the age of 74, Monet only painted summer subjects in his garden and during the winter months he worked in his studios retouching his works and finishing his canvases [49], but he was then diagnosed with a cataract in his right eye and eventually would come to affect both eyes. Despite his visual impairments, Monet proclaimed in 1920, “*I'm extremely busy with my garden; it's such a joy to me, and on fine days like those we've had recently I am in raptures at the wonders of nature.*” Monet continued to paint but his deteriorating eyesight caused him severe problems in distinguished colors for many of his final years [49]. While there have been many theoretical arguments about whether the change in Monet's paintings of his garden was due to “late style” or specifically due to limitations in his vision, Marmor used medical knowledge and computer simulation to investigate the impact of visual disabilities on perception. Marmor [50] demonstrated how Monet (and the painter Degas) had their perceptions of their preferred scenes or subjects changed due to disease, which affected their style of painting [51–53]. And for Monet, the loss of color perception due to his complications with cataracts was a major problem (see [45, 53, 54]). As Marmor [50] pointed out, Monet used his beloved garden landscape to capture the nuances of color and light, “*but his cataracts severely changed and challenged the marvelous qualities of color in his works*” (p. 1769). Even though different eye pathologies cause different visual limitations, we now know how low vision can affect both the physical and mental well-being of older adults and the ability to function in a variety of activities of daily living (ADLS) and instrumental activities of daily living (IADLS) [55, 56]. Despite these challenges, Monet stayed connected with the enchantment of his garden space, and his paintings, especially of the water lilies, were his sense of legacy to the world—that to care and cultivate the beautiful is to embrace a larger cosmic sense of nature. Another part of his legacy is his ability to engage this belief while facing physical limitations and obstacles.

The conjunction of gardening and art is substantial and includes mediums from painting to literature to film. Readers are encouraged to review the literary companion publication of Simonds [57], Marranca's [58] anthology, and Garmey's edited book [59] for an introduction and extensive review in this form of art. For a more contemporary nonfiction perspective, Arthur Hellyer's *Your Garden Week by Week *[60], Jamaica Kincaid's *My Garden (Book)* [61], Diane Ackerman's *Cultivating Delight: A Natural History of My Garden *[62], Michael Pollan's *Second Nature: A Gardener's Education *[36], Caroline Holme's *New Shoots, Old Tips *[63], Barbara Kingsolver's *Animal, Vegetable, Miracle: A Year of Food Life *[64], and Robert Fenton's [65] critical review in *The New York Review of Books *are highly recommended.

When it comes to dedication to gardening, many people will think of one of the best-selling American nonfiction classics, *Walden* by Thoreau [66, 67]. But while Thoreau had the ability to capture the art and beauty of being connected to the soil, his stay at Walden was relatively short, a two-year experiment into his late twenties, and then he left to pursue other travels. If one wanted to find a more “constant gardener,” across the entire life course and *into the retirement years*, we could better discuss Thomas Jefferson, Governor of Virginia and Vice-president and President of the United States, who was also an avid gardener. We are fortunate to read of his observations and activities in gardening in a publication titled, *The Garden Book* [68], which he began in 1776 and continued until the autumn of 1824, two years before his death at the age of 83. *The Garden Book* is a remarkable account of Jefferson's meticulous note taking on his botanical interests and indicated a devotion to the “culture of the earth.” At the age of 68 years old, in a letter to a Charles Willson Peale written in 1811 [68], Thomas Jefferson remarked that, “*But though an old man, I am but a young gardener.*” And the theme of gardening continues as both therapeutic activity and metaphor for life itself in the works of Hans Christian Andersen; the garden became an allegory for his lifework writing folk tales (“fairy tales”), which had more significance with the world of adulthood than for children. The folk tale, “The Gardener and the Gentry” was written *toward the end of his career* and two years before his death in 1872. The use of gardening as a metaphor and allegory continues in the works of noted writers such as Frances Hodgson Burnett's* The Secret Garden *[69], Charles Baudelaire's *Les Fleurs du mal* [70] and Eliot's imagery of the rose-garden in the *Four Quartets* [71] carries a multilayered meaning of spirituality and the loss of Paradise within the cycles of life and death [72]. The *Four Quartets* were written over a span of several years (1935–1942) and in the last quarter of Eliot's life. In “Burnt Norton” the rose-garden conveys memories and mythology of time passing with human existence (mere moments) compared to the history of humanity, civilization, and all that has gone before. We find it correlative that Carl Jung was using a similar theme to address his understanding of his own life in his book *Memories, Dreams, Reflections *(1989). In 1957, at eighty-one years of age, Jung began to work with Aniela Jaffe to complete this major work before he died in 1961. The enlightening passage is from the prologue and is both vegetative and seminal in its garden metaphor by picturing life individually and collectively as sustaining and regenerative over the ages (see also [73]).

*When we think of the unending growth and decay of life and civilizations, we cannot escape the impression of absolute nullity. Yet I have never lost a sense of something that lives and endures underneath the eternal flux. What we see is the blossom, which passes. The rhizome remains (p. 4).*

Although Jung's use of the rhizome metaphor supports the philosophical impression of life as continuous and unending, despite the relentless seasons and centuries of time, gardens are also very much transitory and impermanent. And so while gardens can be perceived as being artistic [74], they may not necessarily represent the outcome that matches the Hippocratic dictum, “Life is short, art is long” (*Ars longa, vita brevis*) in the sense that a garden is cultivated and cared for in an effort to outlast its caretaker, while on the other hand, gardens exist to reenchant the present [75, 76]. Yet, one long-lasting medium for the expression of art is through the vehicle of film. Here we will examine the crossroads of the use of garden imagery (symbolic and realistic) in relation to the aging experience and focus on two exemplars: *Wild Strawberries and Dreams.*

*Wild Strawberries* is the 1957 film by Bergman [77] that depicts the story of the aged Dr. Isak Borg who is traveling in his car to receive an honorary degree. Erikson [78] has provided a comprehensive analysis, based on his own theoretical interpretation, of Bergman's film and it is here that Erikson sees the emergent virtue of *care* as a necessary strength for “*the life cycle as well as the cycle of generations*” (p. 7). For example, Erikson noted that the tensions found between Dr. Borg and Marianne (his daughter) and Borg's son Evald reflect the core issues of generativity such that it is Borg who must confront his own rejectivity and the resulting lack of care and interest in his own family and many others around him (see also [79]). The turning point for Isak Borg, and obviously the primary inspiration for the title of the film, is when Dr. Borg leaves the main highway (his journey of life) and drives down a side road to revisit an old summer home (a chance for reminiscence and remembrance out of the rigid pattern of living in rote predictability). Borg remembers a specific location that would serve as catalyst for a reawakening much like the Proustian madeleine, but in this case, it is a strawberry patch near the summer home [80]. And it is here that we find a richly layered symbolism that involves the magical transformation of the landscape surrounding the home into blooming plants and trees and lush greenery. Even though the film is in black and white, one can almost imagine that large yard as colorful as Monet's gardens at Giverny. The wild strawberries that grow along the side of the yard are the triggering mechanism to transport Borg back through time and allow him to revisit his own young adulthood in relation to Sara, his “first love.” In this film, the bounty of the earth in the form of wild strawberries is richly symbolic of the decisions made and the missed opportunities at forming intimate relationships (with Sara) and the resulting isolation and aloofness that made him think of himself as a “living corpse.” Erikson [78] offers his analysis of the Arcadian scene,

*“one senses that this whole earthy scene, beyond its precious gaiety and its symbolic reference to defloration, points to something primeval, some garden, long forfeited by Isak” (p. 8). *

At least from Erikson's perspective, that small patch of botanical life, the wild strawberries, is at once symbolic of the epigenetic pathway and a crossroads through adulthood and into the commitments of *mature caring within mid and later life*. This theme shall become an important factor to consider when we examine the role of stewardship as a dimension of the “gray and green” concept for the aging experience.

Kurosawa, at the age of 80, directed and then released the film,* Dreams* [81], in 1990 (his 28th film), which featured several portrayals of Kurosawa's own dreams experienced over the course of his life. The first and last episodes of *Dreams* feature two processions that symbolize the opening and closing of the life cycle: a wedding and a funeral [82]. The film also captures luminous sequences of botanical wonderment and rural scenes of wheat fields (*crows*) with Van Gogh (who also loved gardens; see [83]) at work painting his landscapes and other segments that portrayed the ruins of ecological disasters and the break with nature. The last segment, “The Village of the Watermills” of the film is especially significant which captures a lush farm with blooming flowers, lush green grass, and crystal clear rivers that drive the watermills in a wheel-like fashion. It is a “*paradisiacal place: a village where modern technology has not invaded people's lives and they live in harmony with nature*” [83]. The Kurosawa surrogate (the younger man), while walking through the village, encounters a 103-year-old man, who is working on a smaller water wheel structure. The older man communicates the necessity of treating the land with respect and articulates the perils if it being mistreated. The symbolic depth of the old man imparting wisdom (a representation of Eriksonian “generativity”) to the younger man is a critical link in the message of the segment which highlights that working in harmony with the land helps to create a natural cycle of living, and dying. The watermills turn with the river of water, which provides nourishment for the plants and the flowers, which in turn the villagers use to celebrate the wheel of life, and death. At the end of the film, we are left watching a slow moving river current with undulating clusters of long swirling stands of lush aquatic plants swaying with the flow of water that reflects multiple colors on its surface, blues, greens, and the liquid silver of indirect sunlight. Much like a Monet oil painting, *Water Lilies,* and his Japanese Bridge over his pond.

These artists demonstrate the ability to engage and connect despite challenges—an ideal for any aging adult. However, the process of disengagement seems to contradict the presence of this ability as the individual retreats into himself, and rather than being a constant gardener, he becomes isolated and trapped in his memory and his past. An example of this is Grey Gardens, a documentary so fascinating that it has been translated into a Broadway musical and an HBO film. With the title of Grey Gardens (1976) such a film would appear to be the perfect connecting point for examining aging issues (“grey”) and gardens. But the film is less about gardening in the later years (per se) and more about what was once cared for in relationships with people, home, and landscape in the past has instead fallen into a state of neglect due to the disconnect with cultivating what is alive in the present. Grey Gardens is actually the name of the decaying estate that belongs to Edith Bouvier Beale (“Big Edie”) age 79 and her daughter Edie (or “Little Edie”) in her fifties, both frozen in time and place, in an East Hampton home that is graying along with them [84]. In the early 1970s, their 28-room mansion was found to be a health hazard and mother and daughter were threatened with eviction. The film opens up with Little Edie telling the Maysles about what used to be in terms of the beautiful and exotic gardens that once was [84]. Throughout the film, there are shots of the house surrounded by thickets of vegetation, a landscape “gone wild,” that makes it appear deserted, forlorn, and enveloped in an ivy-snarled ruin. In Grey Gardens there actually is a man who is introduced as the “gardener” at the beginning of the film, but it is clear that the intruding jungle of vegetation is overwhelming to him. As the mother and daughter live in the past, the present landscape is removed from care and attention and nature encroaches and erodes the house. After years of neglect, raccoons move into the attic, ivy presses through window seals, and cats continue to reproduce and turn every room into a new wing of a sprawling litter box. The two women cook, eat, and spend most of their time in the bedroom the two of them share, warming canned goods on a hotplate near Big Edi's bed. Decay envelops what was once lush, beautiful, and a welcoming example of cooperation between nature and human interaction. Harrison [75] succinctly stated the connection between the need for constant gardening in the face of our obligations to the here and now, “If we are not able to keep our garden, if we are not able to take care of our mortal world, heaven and salvation are vain” (p. 11).

Gardening, then, is not just a hobby or a task meant to keep one physically active or to keep the mind occupied. It is effort against entropy. It is the cooperation between nature and humanity, fine-tuned through experience and nurturing. Gardening is age and wisdom.

Wisdom may be gained in the privacy of one's own mind, but it's true worth manifests as wisdom is shared. The artistic examples referenced here are not just means of self-expression, they are bridges from individual understanding to community stewardship and care. The proper starting point for this discussion of art bridging personal wisdom and community should be with Virgil's *Georgics*. Virgil (70 BCE-19 BCE) wrote the poem *Georgics* (the word *Georgics* refers to primarily to “farming”) during the 30s BCE and this would put Virgil at just over 40 years of age, and relatively speaking, at the peak of his senior status in the understanding of the life course at that time but perhaps not *senectus *yet [85]. *Georgics* is a didactic and very much a template and design for interacting with the land and being attentive to what Ferry [86] noted as Virgil's ability to engage the “*ecstatic and tender celebrations of the very life in things,*” and more importantly how these “celebrations” interact with human existence (see also [87]). In the *Georgics*, one can appreciate Virgil's attentiveness to the cycles of seasons, and the cycle of birth and death, and the inevitable unfolding of sickness and aging. Virgil's *Georgics *emphasizes that through *care* of the land, we begin to care for each other, and from there follow the arts and cultural blossoming and the resulting harvesting of another kind: poetry, art, music, sculptures, law, and ethics. In the fourth Georgic, Virgil writes of an old man who is at work on his small patch of land that was at one time not fertile enough to be plowed by oxen, but with his dedicated attention it has been transformed. The lines in this vignette are less didactic and more poetic in the sheer strength of the action and sensuality by use of vivid descriptions of the labor that is needed for all the seasons [88]. Quint [89] has proposed in his review of the new translations of *Georgics* by [86] and Lembke [90] that, “*the old gardener thus carries some of the poem's political hopes as well as its ethical message. From a life of turmoil, he has settled into quiet usefulness and contentment, tamed by work and hardship, and even makes a thing of beauty in his flower garden, an analogue to the poem itself”* (p. 35).

An overarching theme throughout the *Georgics* is the didactic lesson of “as you sow, so shall you reap.” The lesson is that progress towards happiness and well-being is highly dependent on the service that you have rendered to the land, kith and kin, to neighbors and to community. And it all begins in your backyard. Thus, gardening serves as the cornerstone of civilization-that is, *to care*, *constantly*. Harrison [75] has succinctly woven these themes together by stating, “*The true gardener is always ‘the constant gardener*” (p. 7).

### 3.2. Science Discipline: How Gardens and Gardening Increase Stewardship, Community Awareness, and a Connection to Future Generations and Individual Health

The constant gardener cares for and nurtures nature, community, and future generations to come. Gardens not only provide rejuvenation of plant life and the aging individual, but also inject life and vitality into the community. Here, we examine two prime examples of the “gray and green” concept where the “green” effect of gardens and gardening can instill the potential for stewardship toward not only one's backyard, but toward a much larger sphere of environmental concern and care directed “outward” in the form of legacy, community, and welfare for future generations. Gardens and gardening can also provide a direct benefit through the restorative impact of either interacting with and in the garden as a physical activity or that the presence of a garden itself as a sensory experience can enhance well-being and health.

If the lesson from the *Georgics* is that community begins in the backyard, it is no wonder that thoughts turn to residential landscaping and lawn care while thinking of communities and gardens. Lawn care has been the most prevalent form of gardening nationwide. However, this dimension has been going through its own transformation and redefinition as many more people are looking to redefine the “lawn” into a more environmental friendly and regionally appropriate recreational and social site for families, including the expansion of gardens [35, 91]. Brown and Jameton [92] have indicated that there are numerous benefits for the increase and support of gardening: food security and nutritional health (home grown produce has the potential to offset the cost of purchasing food), positive effects on physical health (as exercise), and overall community improvement (to enhance social capital; it can serve as a community organizing tool to combat poverty and provide a collective response to blighted city neighborhoods) and as a way of raising consciousness about environmental stewardship. Brown and Jameton [92] also suggest various community-based policy recommendations to encourage urban garden activities because, “*Urban gardening raises our public awareness of the need to safeguard our environment, and especially our urban soils, from future pollution, erosion, and neglect*” (p. 33). More specifically to older adults, Ashton-Shaeffer et al. [10] discovered many motivational factors for gardening in their investigation. For example, they found significant differences among older adults by marital status, education, and health status in terms of motivational categories. The two most important categories were physical fitness and creativity. Similarly, Wang and Glicksman [93] reported that low-income minority older adults expressed several motivations and benefits of community gardening. For example, Wang and Glicksman identified nine themes of why older adults chose to participate in gardening: mental health benefits, the end product (fruits and vegetables), continuation of a past life, something to do/responsibility, beauty and connection to growth, connecting with others, physical health, learning something new, and helping each other out [93].

Fänge and Ivanoff [94] also discovered mixed results with gardening in men and women aged 80–89 in Sweden. Fänge and Ivanoff indicated that while gardens provided opportunities to go outdoors and offered a meditative space for older adults, having a garden to take care of could be a “considerable practical burden and was something that bothered the very old peoples' minds” (p. 342). In contrast, Pettigrew and Roberts [95] have proposed in their study that gardening had served as an effective way for older adults to ameliorate the experience of loneliness and feelings of emotional isolation. Gardening was also identified as a mechanism to help facilitate “self-reconstruction” through spatiotemporally establishing biographical continuity between older Chinese immigrants in their new lives in New Zealand [91].

We also know that gardening can serve as a “bridge-building” activity for enhancing intergenerational cooperation in community settings [96–100] and that it can represent a form of legacy in older adults [101] and serve as a mechanism to engage in “successful aging” [102].

The phrase “successful aging” recollects Monet and his ability to continue producing art despite his failing eyesight. The aging process requires adaptation and the ability to approach tasks in a different, more sustainable way. There are research findings to indicate gardening as an activity to enhance the physical and emotional well-being for older adults who reside in home and community-based dwellings [103]. For example, Infantino [104] found that the gardening experience had sustained older women in their cognitive and spiritual development. Heliker et al. [105] found that horticultural projects (consisting of 12 weeks of interactive gardening classes) were instrumental in increasing a sense of psychological well-being in racial and culturally diverse groups. They also found that gardening helped to instill a deeper sense of legacy and spirituality and a deeper relationship with the earth and nature in the older participants. Quandt et al. [106] explored the nutritional role of “food sharing” among a diverse set of older rural adults and also highlighted the “social meaning” in such activities as well. Similarly, Milligan et al. [107] found that older adults benefited from gardening in communal allotments as it helped to overcome social isolation and contributed to the development of social networks. An additional benefit of affective restoration from stress was found among adults (mean age 57.6) in an experimental study comparing gardening to indoor reading [108]. The authors noted that gardening may permit people to enjoy the restorative effects of nature on a regular basis. In another related study, Hawkins et al. [109] discovered that allotment gardeners appreciated “doing” gardening activity as well as “being” at the allotment landscape for affording a wide range of benefits to their health and being, specifically in stress reduction.

Gardening as an activity to improve the quality of life for older adults has generated a substantial number of publications that address gardening as a physical activity for older adults that may facilitate healthy aging [14, 15, 110–112] and as an intervention with *indoor* gardening and horticultural therapy for institutional populations [15, 113–129]. The intersection of gray and green is also reflected in the paradigmatic shift of “The Eden Alternative” in managing long-term care facilities [130, 131]. For example, Lee and Kim [132] found that indoor gardening was found to be effective for improving several dimensions of sleep patterns, decreasing agitation, and improving cognition functioning of dementia patients (see also [133]). There are also significant publications on *outdoor* gardening activities for people who live in geriatric care settings [134], specifically for persons with dementia [135], and the importance of “patient-specific gardens” for the restorative effects of nature [136]. Goto et al. concluded that organized gardens can positively affect both mood and cardiac physiology of elderly individuals [137]. Gardening has also been identified as a potential activity to incorporate into fall prevention programs as gardeners reported significantly better balance and gait speed and had fewer chronic conditions and functional limitations than nongardeners [138]. However, both Kwak et al. [139] and Larner [140] have also suggested that many cognitive abilities may be required for successful gardening and horticultural activities and that if gardening is being considered as a component for intervention and therapy for dementia patients, an individual approach that is customized to abilities and deficits and the various symptoms may be required.

In Valliant's [141] monumental study of aging and well-being, *Aging Well, *he ended with a chapter titled, “Positive Aging: A Reprise,” and in that section there was the creative metaphor that captured the nuances of growing older as akin to gardening or, more precisely, the lessons learned in *being* a gardener could serve as a positive role model for finding fulfillment in later life.

Among the many positive attributes, Valliant proposed that gardening is an activity that encourages a therapeutic *slowness* [142, 143] and brings with it the additional benefit of creating opportunities for introspection and reflection (that you should stop and “smell the roses”) and that it encourages and facilitates the overarching Eriksonian concept of *care*: a hallmark and defining positive attribute of the aging process [144]. We are reminded of Shakespeare's lament, “*sweet flowers are slow and weeds make haste*” which all but exudes the wisdom of living a long and cultivating the rewards of a life well-lived *and cared for*. Valliant also proclaimed that gardeners are very much in the spirit of generativity and symbolically working (i.e., being concerned and having responsibility); the soil is embedded with the meaning of rebirth and regeneration, stewardship, and the essence of cultivating for the next cycle of life [145]. And with this kind of care, there is the potential for the legacy of *caritas*, a *vita activa*, and a vocation of care transmitted through the generations. Thus, as Valliant succinctly states it, “*There is a kind of immortality about gardens, at least until next spring and the spring after that*” (p. 309).

Not only does Valliant make reference to Cicero and the ancient Roman tradition of viniculture as an honorable activity for aging, he revisits the famous line from Voltaire's *Candide* [146] where after many journeys, hardships and mishaps and a great deal of theorizing, Candide instructs Pangloss, “*We should cultivate our gardens*.” That pithy philosophical statement was crafted by Candide and inspired from an earlier encounter with an old man who was sitting outside his house “*minding his own business*” and taking in the day and enjoying the “*fruits of his labor*.” This encounter sounds very familiar to a similar story of the old man previously mentioned in Virgil's *Georgics*.In Voltaire's novel, the old man knew little of worldly affairs and events but graciously offered Candide, Pangloss and Martin a sumptuous meal of exotic fruits and nuts that were grown on his farm. Candide assumed the old man must have some vast and magnificent estate, but the old man said, “*I have only twenty acres and I cultivate them with my children; and my work keeps at bay three great evils: boredom, vice and need.*” It took a while, but Candide had suddenly become a fast learner. *Gardening?* Yes, of course. Candide thought it to be a far better existence compared to anything else they had encountered as history unfolded not too kindly in all of their travels and experiences.

Perhaps it is best to summarize the findings of the importance of gardening in the lives of older adults by highlighting the work of Bhatti [11] who found that the presence of and the interaction with gardens can have a major significance in the (re)creation of “home” in later life. In addition to the benefits of physical activity [13–15, 110, 147] there is the added dimension of what the garden symbolizes psychologically as a meaningful reason for existence, or as one older adult expressed it, “*when I'm in the garden I can create my own paradise.*”

Here, we have come full circle. We began with the sensory qualities of gardens and gardening and the personal spirit-mind-body benefits to cooperation between man and nature. Personal well-being often translates into artistic expression, whether it is through painting, literature, film, or the act of gardening itself. This artistic expression benefits communities and creates a legacy for the aging adult while providing physical and mental health benefits. The give-and-take between nature and individual, individual and community, and community and nature is ongoing and rejuvenating. Science and the humanities continue in a symbiotic relationship just as nature and human beings do, and this cooperation is vital to the well-being of aging individuals and of every human being at any stage of life.

We should cultivate our gardens, but what is it exactly about gardens and gardening that would supply meaningful significance to the life course, and particularly to the aging process? What is the connection between gardening and care and well-being? The analysis of the scientific validity of the health benefits of gardening for older adults has been aptly analyzed by Wang and MacMillan [15] and they correctly conclude that there is a need for larger-scale studies using probability samples and combined methodologies to develop a more comprehensive understanding of the nuances with gardening and the aging experience. While the systematic review of Wang and MacMillan focused on the merits of gardening in the domain of connections to family and community and cultures, we would submit that the additional *connection to the natural environment*—the gray and green concept—is worthy of sustained investigation as well, especially as it relates to larger macrodynamics of legacy, sustainability, generativity, and intergenerational support and caring [148, 149].

## 4. Synopsis and Conclusions to the Review Paper

The purpose of this paper was to review several multidisciplinary nodes of gardening and the aging experience and to examine two specific issues: (a) gardening as a significant activity to engage the cultivation of caring across the life course, and (b) as a way to enhance the notion of stewardship in supporting environmental health in the context of home and community based settings. Based on the review of the selected literature and the examination of various multidisciplinary perspectives, we can propose that gardens and gardening represent multidimensional phenomena in the lives of many older adults and it is much more than a physical activity in a designated space where time and energy are exerted for cultivating fruits and vegetables. Gardens and gardening can also represent an intimate connection with life itself through caring and being a steward for living organisms that also reciprocate with nourishment, aesthetics, and existential meaning in the context of senescence. As Roszak [150] has noted, the added dimension of environmental connection and awareness into the experience of the aging process could serve as a template for a new “elder culture” and a sustainable future.

In addition, there are the beneficial therapeutic interactions with gardens as healing spaces, as in the example of “great-granny's garden” (Oslo, Norway), which not only is represented as a living archive for botanicals, but also serves as a “sensory garden” for persons with dementia [151]. This captures an excellent concept for both conservational activities *and* supporting the therapeutic goal of the positive attributes of a garden space for older adults with cognitive impairments.

There is much to discuss within a synopsis of these themes, but one link to appreciate is the connection back to Valliant's synthesis of his research findings that point to gardening as including, but much more than,* activity* of physical effort, and rather, it takes on the gravity of a philosophy of life, a *Weltanschauung*, a raison d'être, and much more. Valliant indicated that instead of the all-elusive high expectations of *happiness *to be sought for in later life, the concept that might instead be a better fit given the realism of the aging process in terms of challenges and promise is a *joy in life*. The experience of a joyful attention to life (and a life) that is cultivated in gardening is very much akin to a meditative practice [152] and helps to create a new kind of “homecoming” that allows one to become native to a place where there is a deeper connection to seasons of growth, and decline [153, 154]. To many, the garden exemplifies a new agrarian standard [155] that integrates the many realms of the “Great Garden” that is a seamless gateway between many realms of nature including trees, streams, pastures and it helps to instill a redirecting of *mindful care* [156].

Before you might think that the topic of gardening is exclusively an opportunity for those who are geographically located in rural areas or along the exurban fringes, and thus beyond the reach for urban and inner city dwellers, consider the following example. In the low-income residential neighborhood geographically located in the Bronx (New York) known as Tremont, there exists a community garden that recently celebrated its 37th anniversary. And it is here that the residents of Tremont find a sense of belonging even while surrounded in a world of concrete. Tina Kelley wrote a story for *The New York Times* on the Tremont Community garden and said that the “*gardens are oases, where a collective spirit and a sense of community grow from the topsoil*.” The President of the community garden is Elizabeth Butler (age 77) and she said that the garden is like a refuge, “*If I couldn't come here, it would be rough … I cannot stay in the house*.” Another garden member, a retired nurse, said the garden is site for celebrating birthdays and as a place for memorial services as well. “*They were like deeply part of the garden, like a soul thing*,” while another community member said of the garden, “*It's my joie de vivre. I like the way it looks. I enjoy the view. I sit here by myself*.”

*Joie de vivre* means joy of life.

And then there is Candide: *Il faut cultiver notre jardin*. Yes, we should cultivate our gardens. But listen to how Harrison [75] has interpreted *notre jardin* from Voltaire's story,

*Notre jardin* is never a garden of merely private concerns into which one escapes from the real; it is that plot of soil on the earth, within the self, or amid the social collective, where the cultural, ethical, and civic virtues that save reality from its own worst impulses are cultivated. Those virtues are always *ours* (p. x).

It may be difficult to imagine this much ado about a patch of soil, some seeds, watering, and having a “strong back and a weak mind” with all the weeding and mulching [157]. But Voltaire was onto something. And that “something” has resonated with many older adults throughout history and is today still both a viable and contemplative activity that is both elemental and transcendental. That “something” is related to the core virtue of the mature and responsible adult in mid and later life: *the ethical basis of care* [144, 145]. The caring for the garden (local) is then extended into the caring of something *beyond* the home and community and outward into social and cultural levels (global) [158]. This is exactly the link that Collins [159] discovered in her study of community dwelling older adults who were transformed into “keepers of the earth” through the activities of a “gardening life” and as a result, environmental stewardship was facilitated. It is our conclusion, that the ultimate expression of why the act of cultivating is so compelling in the later years of life is connected with Stanley Kunitz who spent a lifetime (over a hundred years) reflecting on the importance of gardens through poetry. In the book, *The Wild Braid* [76], the nexus of cultivation, caring, and the life course are poignantly discovered,

*I think of gardening as an extension of one's own being, something as deeply personal and intimate as writing a poem. The difference is that the garden is alive and it is created to endure just the way a human being comes into the world and lives, suffers, enjoys, and is mortal. The lifespan of a flowering plant can be so short, so abbreviated by the changing of the seasons, it seems a compressed parable of human existence (p. 14). *

The opportunity to consider the benefits of gardening in the aging process as including a viable connection to outcomes relating to stewardship and care and as a part of the process of older adults being engaged with natural contexts beyond the physical and built environment is also the possibility of further investigating the gray and green phenomena as an important dimension in well-being and optimal aging in mid and later life [1, 3, 4, 93, 148, 160–162]. To be generative, to cultivate, to care is both utilitarian and sacred and finds its joyful expression in the reverential duty in the garden.
